# Acquired Venous Malformation of Bilateral Upper Eyelids

**DOI:** 10.1002/ccr3.71300

**Published:** 2025-10-21

**Authors:** Airi Kimura, Yuki Mizutani, Keiichi Yamanaka

**Affiliations:** ^1^ Department of Dermatology Mie University Graduate School of Medicine Tsu Mie Japan

**Keywords:** acquired venous malformation, Doppler ultrasonography, skin biopsy, upper eyelid

## Abstract

An elderly female presented with a two‐year history of bilateral erythematous swellings on the upper eyelids. Doppler ultrasonography demonstrated a low‐flow vascular pattern. Skin biopsy revealed vascular hyperplasia characterized by slit‐like irregular vessels. Based on these findings, a diagnosis of acquired venous malformation of the bilateral upper eyelids was established.

A 74‐year‐old woman presented with a bilateral, medial upper eyelid lesion characterized by elastic, soft, erythematous to yellowish infiltrates that had developed over the preceding 2 years, with a slight tendency to enlarge. The lesions were asymptomatic, with no associated pain or bleeding. There was no history of venous obstruction, trauma, radiation exposure, or chronic inflammation. Initially, eyelid xanthoma was suspected, and treatment with lipid‐lowering agents was initiated; however, no clinical improvement was observed. The patient had no underlying metabolic disorders such as hypercholesterolemia, diabetes mellitus, or hypothyroidism.

On physical examination, a well‐demarcated, elevated, erythematous lesion with elastic‐soft consistency, measuring approximately 10 mm in diameter, was observed on the medial aspect of each upper eyelid (Figure 1A). Doppler ultrasonography revealed increased blood flow with a low‐flow pattern (Figure 1B). Skin biopsy demonstrated diffuse vascular proliferation extending from the mid to deep dermis, composed of slit‐like, irregular, thin‐walled vessels lined by flattened endothelium (Figure 1C). Xanthelasma palpebrarum was excluded because no lipid‐laden foam cells were present in the dermis. IgG4‐related orbital disease, including Mikulicz disease, was ruled out as dense lymphoplasmacytic infiltrates with storiform fibrosis and numerous IgG4‐positive plasma cells were absent. Angiosarcoma was also excluded due to the lack of atypical endothelial cells with multilayering and mitotic activity. Based on the clinical, imaging, and histological findings, a diagnosis of acquired venous malformation (VM) of the bilateral upper eyelids was established. Treatment of VM varies depending on symptoms and lesion size, and sclerotherapy or surgical excision is performed in cases where symptoms or cosmetic issues are present [1]. The patient opted for observation and regular follow‐up.

**FIGURE 1:**
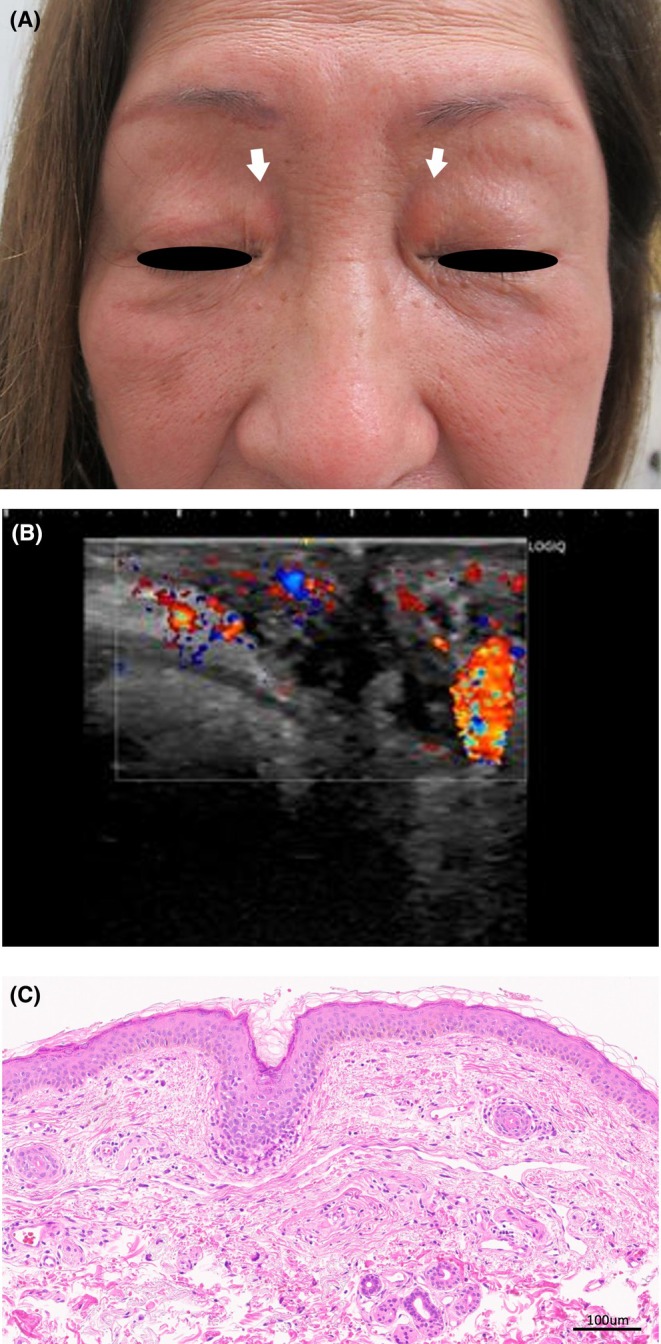
(A) A flat, erythematous, elevated lesion with well‐defined, elastic‐soft borders measuring approximately 10 mm in diameter on the medial aspect of the bilateral upper eyelids. No surface telangiectasia was observed. (B) Doppler skin ultrasonography revealed increased vascularity with a low‐flow pattern. (C) Skin biopsy of the upper eyelid lesion demonstrated diffuse vascular proliferation composed of slit‐like, irregularly shaped, thin‐walled vessels extending from the mid to deep dermis. The vessels exhibited mature luminal structures without endothelial cell atypia. No inflammatory cell infiltration, lipid deposition, or foam cells were present.

VM is the most common type of congenital vascular malformation, typically presenting as a localized, elastic, and firm subcutaneous mass, which may be slightly elevated and exhibit a bluish discoloration or telangiectasia on the overlying skin [1]. VM is typically a congenital, non‐involuting lesion that may enlarge in response to trauma or hormonal changes. Acquired, bilateral, and multiple lesions are extremely rare [2, 3]. However, the use of Doppler ultrasonography and skin biopsy may facilitate accurate diagnosis in such atypical presentations. Written consent for publication was obtained from the patient.

## Author Contributions

**Airi Kimura:** conceptualization, investigation, writing – original draft. **Yuki Mizutani:** conceptualization, data curation, writing – original draft. **Keiichi Yamanaka:** conceptualization, data curation, investigation, validation, writing – original draft, writing – review and editing.

## Ethics Statement

The research was conducted in accordance with the Declaration of Helsinki. The patient gave us consent for her photographs and medical information to be published in print and online, with the understanding that this information is publicly available. The paper is exempt from ethical committee approval due to its single‐case study nature.

## Conflicts of Interest

The authors declare no conflicts of interest.

## Data Availability

The patient's data is not publicly available on legal or ethical grounds.
